# N6-methyladenosine modification of LATS2 promotes hepatoblastoma progression by inhibiting ferroptosis through the YAP1/ATF4/PSAT1 axis

**DOI:** 10.7150/ijbs.92413

**Published:** 2024-08-01

**Authors:** Guoqing Zhu, Yi Xie, Zhixuan Bian, Ji Ma, Ni Zhen, Tianshu Chen, Jiabei Zhu, Siwei Mao, Xiaochen Tang, Li Liu, Song Gu, Miao Ding, Qiuhui Pan

**Affiliations:** 1 Clinical Laboratory, Shanghai Children's Medical Center, School of Medicine, Shanghai Jiao Tong University, Shanghai 200127, P. R. China.; 2 Department of Surgery, Shanghai Children's Medical Center, School of Medicine, Shanghai Jiao Tong University, Shanghai 200127, P. R. China.; 3 Faculty of Medical Laboratory Science, College of Health Science and Technology, Shanghai Jiao Tong University School of Medicine, Shanghai, China; 4 Shanghai Key Laboratory of Clinical Molecular Diagnostics for Pediatrics, Shanghai 200127, P. R. China. Address: Dongfang Road No. 1678, Pudong New District, Shanghai 200127, P. R. China.; 5 Sanya Women and Children's Hospital Managed by Shanghai Children's Medical Center, Sanya 572000, P. R. China.

**Keywords:** hepatoblastoma, ferroptosis, LATS2, Hippo/YAP, m6A methylation

## Abstract

Ferroptosis has attracted extensive interest from cancer researchers due to its substantial potential as a therapeutic target. The role of LATS2, a core component of the Hippo pathway cascade, in ferroptosis initiation in hepatoblastoma (HB) has not yet been investigated. Furthermore, the underlying mechanism of decreased LATS2 expression remains largely unknown. In the present study, we demonstrated decreased LATS2 expression in HB and that LATS2 overexpression inhibits HB cell proliferation by inducing ferroptosis. Increased LATS2 expression reduced glycine and cysteine concentrations via the ATF4/PSAT1 axis. Physical binding between YAP1/ATF4 and the PSAT1 promoter was confirmed through ChIP‒qPCR. Moreover, METTL3 was identified as the writer of the LATS2 mRNA m6A modification at a specific site in the 5' UTR. Subsequently, YTHDF2 recognizes the m6A modification site and recruits the CCR4-NOT complex, leading to its degradation by mRNA deadenylation. In summary, N6-methyladenosine modification of LATS2 facilitates its degradation. Reduced LATS2 expression promotes hepatoblastoma progression by inhibiting ferroptosis through the YAP1/ATF4/PSAT1 axis. Targeting LATS2 is a potential strategy for HB therapy.

## 1. Introduction

Hepatoblastoma (HB) is the predominant pediatric liver malignancy and originates from abnormal stem cells and hepatic epithelial progenitor cells, accounting for 67-80% of all pediatric liver cancers 1, 2. The incidence of HB has increased, which is speculated to be due to the increased survival rates of infants born prematurely or with low birth weight 3. Despite improvements in surgical resection and chemotherapy, patients with aggressive HB still have an unfavorable prognosis due to high rates of metastasis and recurrence 4. Thus, it is critical to explore the molecular mechanisms underlying HB and develop clinical therapeutic strategies.

The latest version of the hallmarks of cancer identifies nonmutational epigenetic reprogramming as one of the 14 cancer features 5. As a branch of epigenetic reprogramming, N6-methyladenosine (m6A) is the most prevalent mRNA modification in eukaryotic cells and regulates various aspects of RNA metabolism, such as translation, splicing, stability, and localization 6, 7. The enzymes responsible for initiating m6A modification are referred to as the “writer” complex and include methyltransferase-like 3 (METTL3) and other subunits, whereas the fate of target mRNAs is determined by “readers,” including IGF2 mRNA-binding proteins (IGF2BP2s) and YTH domain family proteins (YTHDF1/2/3, YTHDC1/2) 8, 9. m6A methylation affecting target mRNAs is implicated in various human diseases, especially cancers 10. Our previous study demonstrated elevated m6A modification and METTL3 expression and illustrated the characteristics of m6A modification in HB 11. However, the underlying mechanism remains largely unknown.

Ferroptosis is a newly identified form of programmed cell death characterized by the iron-dependent accumulation of lipid peroxides 12. In recent years, ferroptosis has attracted extensive interest from cancer researchers due to its considerable potential as a target in cancer therapy 13. Ferroptosis is dependent on antagonism between the accumulation of lipid peroxides and cellular antioxidant systems. Reduced glutathione (GSH) is the major cellular antioxidant and is synthesized from cysteine, glycine, and glutamate 14. Decreasing GSH production or inhibiting glutathione peroxidase 4 (GPX4) activity directly leads to ferroptosis 15. The interaction between the Hippo pathway and ferroptosis has recently garnered considerable attention 16. The Hippo pathway is an evolutionarily conserved cascade involved in various cancers 17. Typically, the Hippo pathway, which consists of macrophage stimulating (MST) 1/2, large tumor suppressor kinase (LATS) 1/2, and the adaptor protein Salvador family WW domain-containing protein (SAV) 1, phosphorylates and inhibits the downstream effector Yes-associated protein (YAP1) 18. Dysregulation of these kinases directly alters YAP1 expression. Numerous studies have demonstrated the oncogenic role of YAP1 during HB proliferation 19, 20. However, few reports have investigated the role of LATS2 in HB.

In the present study, we aimed to comprehensively elucidate the regulatory network of LATS2 that controls ferroptosis in HB. We revealed that the m6A modification of LATS2 mRNA, which YTHDF2 then recognizes, decreases LATS2 protein expression, activating the Hippo pathway. Activated YAP1 expression facilitates GSH synthesis by increasing cysteine and glycine levels via PSAT1. Moreover, LATS2 expression was significantly downregulated in HB tissues and negatively correlated with HB patient prognosis. Our findings provide new insights into the treatment and diagnosis of HB.

## 2. Materials and methods

### 2.1 Patients and tissue sampling

A total of 36 pairs of HB tissues and adjacent nontumor tissues were collected from patients who underwent HB surgery at Shanghai Children's Medical Center from September 2018 to June 2022. This study was approved by the Ethical Committee of Shanghai Children's Medical Center of Shanghai Jiao Tong University of Medicine. All patients provided verbal and written consent.

### 2.2 Cell culture and transient transfection

HepG2, HUH6, HEK293T, and QSG7701 cells were obtained from the Cell Bank of the Chinese Academy of Science (Shanghai, China). HepG2 cells were cultured in minimum Eagle's medium (MEM), HUH6 and HEK293T cells were cultured in Dulbecco's modified Eagle's medium (DMEM), and QSG7701 cells were cultured in Roswell Park Memorial Institute (RPMI) 1640 medium supplemented with 10% fetal bovine serum (FBS) and 1% antibiotics (Gibco, Carlsbad, CA, USA). All cultures were maintained in a humidified 5% CO_2_ incubator (Thermo Fisher Scientific, Waltham, MA, USA) at 37°C. For transient transfection, HepG2 and HUH6 cells were seeded at a density of 2.5 × 10^5^ cells/ml in 6-well plates and incubated overnight until they reached 40-60% confluence. The cells were transfected with LATS2 and YAP1 plasmids (GenePharma, Shanghai, China) using Lipofectamine 2000 (Invitrogen, Carlsbad, CA, USA) and incubated for 48 h. The target sequences are listed in Supplementary Excel 2-S2.

### 2.3 Plasmid transfection and lentivirus transduction

HepG2 and HUH6 cells were transfected with plasmids using ExFect2000 Transfection Reagent (Vazyme, Jiangsu, China) according to the manufacturer's instructions. The plasmids mentioned above and a packaging plasmid were transfected into HEK293T cells for lentiviral transduction. After 48 h, the supernatants were collected and used to infect HB cells for 48 h. Finally, the HB cells were cultured in the presence of 2 μg/ml puromycin to obtain stable cell lines.

### 2.4 Immunohistochemistry

Paraffin-embedded sections were deparaffinized using xylene and hydrated with an ethanol gradient. Subsequently, the sections were incubated in 3% methanol-H_2_O_2_ and transferred to a 0.01 mol/L sodium citrate solution for antigen retrieval. Next, the sections were blocked with 5% skim milk powder solution at 25°C and then incubated first with anti-LATS2 (1/100; Proteintech, 20276-1-AP) or anti-YAP1 (1/200; Abcam, ab52771) antibodies and then with secondary antibodies (1/1000; Abcam, ab7090). DAB was used to develop the color, and neutral gum was used to mount the film.

### 2.5 Western blotting assay

Cells were lysed on ice using RIPA lysis buffer (Beyotime, Jiangsu, China) supplemented with phosphatase and protease inhibitors. Total protein was quantified, and the samples were boiled in SDS. Afterward, 60 μg of protein samples was separated on an SDS‒PAGE gel and electrophoretically transferred onto nitrocellulose membranes (Millipore, Bedford, MA, USA). The membranes were subsequently incubated overnight with primary antibodies at 4°C. Then, the membranes were washed and incubated with fluorescently conjugated secondary antibodies (LICOR, Tucson, Arizona, USA). Finally, the bands on the membranes were visualized using an Odyssey instrument (LICOR). The primary antibodies used are listed in Supplementary Excel 2-S1.

### 2.6 Confocal immunofluorescence (IF) assay

HB cells were seeded into 24-well plates with coverslips. After treatment, the cells were fixed and blocked with 5% bovine serum albumin. The cells were then incubated with antibodies against YAP1 (1/200; Abcam, ab52771) and ATF4 (1/100; Proteintech, 60035-1-Ig) at 4°C overnight. The next day, the cells were washed with phosphate-buffered saline (PBS) and incubated with Alexa Fluor 488-labeled or Alexa Fluor 647-labeled secondary antibodies (Beyotime, Jiangsu, China). Then, the cells were stained with 4,6-diamidino-2-phenylindole (Invitrogen, Thermo Scientific, Shanghai, China). Finally, the slides were analyzed using a Leica TCS SP8 confocal fluorescence microscope.

### 2.7 Cell proliferation and colony formation assay

Cell viability was assessed using a cell counting kit-8 (CCK8) reagent (Beyotime, Jiangsu, China). In brief, the cells were seeded into 96-well plates and treated as indicated. Subsequently, 100 μl of fresh medium was added to each well containing 10 μl of CCK8 solution and incubated for 2 h (37°C, 5% CO_2_). Afterward, the absorbance was measured spectrophotometrically at 450 nm. The collected values were normalized to the absorbance of the blank wells, and the relative cell viability was normalized to that of the respective DMSO-treated wells. For colony formation assays, the cells (500 cells/well) were seeded in 6-well plates and incubated for 1-2 weeks until cell colonies appeared; then, the cells were fixed with methanol and stained with Giemsa.

### 2.8 Cell apoptosis analysis

After treatment, approximately 5 × 10^5^ cells were collected and washed. Subsequently, the cells underwent staining with FITC-conjugated Annexin V (eBioscience, California, USA) for 10 min, which was followed by staining with propidium iodide (PI) (eBioscience, California, USA) for additional 5 min. Finally, the percentage of apoptotic cells was ascertained using a FACS flow cytometer (BD, New Jersey, USA).

### 2.9 Xenograft tumor assay

The animal research in this study was approved by the Shanghai Children's Medical Center. A xenograft tumor model was established by injecting 1 × 10^7^ cells subcutaneously into 4-week-old male nude mice. Each group consisted of five mice. After 28 days, the mice were sacrificed, and the tumors were weighed.

### 2.10 Lipid ROS assay

The cells were cultured in a 6-well plate and incubated with 5 μM BODIPY-581/591 C11 (D3861, Thermo Fisher Scientific) for 30 min (37°C, 5% CO_2_), which was added to the culture media. The cells were harvested using trypsin without EDTA, washed two times with PBS, and resuspended in 500 μl of PBS. ROS levels were measured using a CytoFLEX cytometer (Beckman Coulter, California, USA), and the data were analyzed using CytExpert software (Beckman Coulter, California, USA).

### 2.11 MDA, iron, and GSH/GSSG assays

The malondialdehyde (MDA) concentration was assessed using a lipid peroxidation assay kit (Abcam, ab118970). GSH and GSSG assay kits (ABclonal, RK05819) were used to measure the GSH/GSSG ratio. The intracellular iron level was determined using an iron assay kit (Abcam, ab83366).

### 2.12 Glutamine, cysteine, phospholipid, and glycine assays

Glutamine concentrations were measured using a GLN1 Kit (Sigma, Saint-Quentin-Fallavier, France). The cysteine, phospholipid, and glycine concentrations in the cells were measured using cysteine (Sangon, D799572-0100), phospholipid (Abcam, ab234050), and glycine (Abcam, ab211100) assay kits.

### 2.13 RNA extraction and quantitative reverse transcription polymerase chain reaction (qRT‒PCR) analysis

Total RNA was extracted from the HB tissue and HB cell lines using TRIzol reagent (Invitrogen, Thermo Scientific, Shanghai, China). Subsequently, total mRNA was reverse-transcribed into cDNA using a PrimeScript reverse transcription reagent kit (TaKaRa Bio, Shiga, Japan). Relative expression was determined by qRT‒PCR using a SYBR Green reagent kit (TaKaRa Bio, Shiga, Japan) in an ABI 7500 PCR system (Applied Biosystems, Forster City, California, USA). The 2^-ΔΔCT^ method was applied for relative quantification, and Pearson's correlation analysis was performed using the ΔΔCT method. Gene expression was normalized to that of 18S rRNA. The primers used are listed in Supplementary Excel 2-S3.

### 2.14 Dual-luciferase reporter assay

HepG2 and HUH6 cells were seeded into 24-well plates and incubated for 24 h until they reached 60-70% confluence. Psi-LATS2-3′-UTR WT and Psi-LATS2-3′-UTR mutant reporter plasmids were constructed in advance. A Dual-Luciferase Reporter Assay System (E1910, Promega, USA) was used to detect firefly and Renilla luciferase activities, which were recorded using a GloMax 96 Microplate Luminometer (Promega, USA).

### 2.15 Chromatin immunoprecipitation (ChIP) and ChIP‒qPCR

The ChIP assay was performed using the ChIP Kit Magnetic-One Step (ab156907, Abcam) according to the manufacturer's instructions. Purified DNA was analyzed by qRT‒PCR with a SYBR Green reagent kit (TaKaRa Bio, Shiga, Japan) following the manufacturer's protocol. The ChIP‒qPCR primers used are shown in Supplementary Excel 2-S3.

### 2.16 Methylated RNA immunoprecipitation (MeRIP)

Cells with stable knockdown of METTL3 and stable overexpression of LATS2 (WT/MUT) were subjected to a MeRIP assay using a Magna MeRIP™ m6A Kit (17-10,499, Millipore, MA) following the manufacturer's instructions. m6A modification of particular genes was determined by qRT-PCR analysis with specific primers. We focused on the potential m6A sites in the 5′ UTR near the stop codon and designed primers to ensure that the target sequence contained all these sites with a limited length of 100 nt. All primers for MeRIP-qPCR are listed in Supplementary Excel 2-S3.

### 2.17 Rapid amplification of cDNA ends-poly(A) test (RACE-PAT)

The length of the poly(A) tail of the LATS2 mRNA was measured using RACE-PAT. Briefly, total RNA was reverse-transcribed with an oligo(dT) primer linked to an oligo(dT) anchor (the primers are shown in supplementary Excel 2-S3). Next, PCR amplification was performed with the LATS2 forward RACE primer and the oligo(dT)-anchor PCR primer, yielding a mixture of PCR-amplified products representing the length of the LATS2 poly(A) tail. PCR products were resolved on a 1.5% agarose gel.

### 2.18 RNA pulldown assays

Partial LATS2 5'UTR probes with adenine (A) and m6A modifications at the putative GGAC motif and a negative control were synthesized and labeled with biotin (Sangon Biotech, Shanghai, China). The detailed sequences are listed in Supplementary Excel 2-S4. For the pulldown assays, the proteins extracted from HepG2 cells were incubated with 3 μg of biotinylated probes on a rotating wheel at 4°C overnight. Then, the RNA‒protein complexes were incubated with streptavidin-conjugated beads (Thermo Fisher Scientific, Waltham, MA, USA) at 4°C for 4 h. The beads were washed three times, boiled, and subsequently analyzed by gel electrophoresis.

### 2.19 Reagents and concentrations used

Ferrostatin-1 (S7243), Z-VAD-FMK (S7023), and necrosulfonamide (S8251) were purchased from Selleck Chemicals (Houston, Texas, USA). Actinomycin D (HY-17559) and cycloheximide (CHX; HY-12320) were purchased from MedChemExpress (New Jersey, USA). The stimuli used were as follows: ferrostatin-1, 2 μM; Z-VAD-FMK, 10 μM; necrosulfonamide, 0.5 mM; actinomycin D, 5 μg/ml; and cycloheximide, 0.1 mg/ml.

### 2.20 Statistical analysis

In this study, all the statistical analyses were performed using SPSS software or GraphPad Prism 8. Continuous variables were compared using appropriate parametric tests. Unpaired Student's t-tests were used to compare the means of two groups, and one-way analysis of variance (ANOVA) was used to compare multiple groups. The chi-squared test was used to analyze the association of the expression levels of LATS2 with clinicopathological characteristics without adjusting for any relevant factors. Unless otherwise stated, a significant difference was considered when the following p values were obtained: * p <0.05, ** p <0.01, *** p <0.001, or **** p <0.0001.

## 3. Results

### 3.1 Decreased expression of LATS2 facilitates HB proliferation by inhibiting ferroptosis

**Figure 1:**
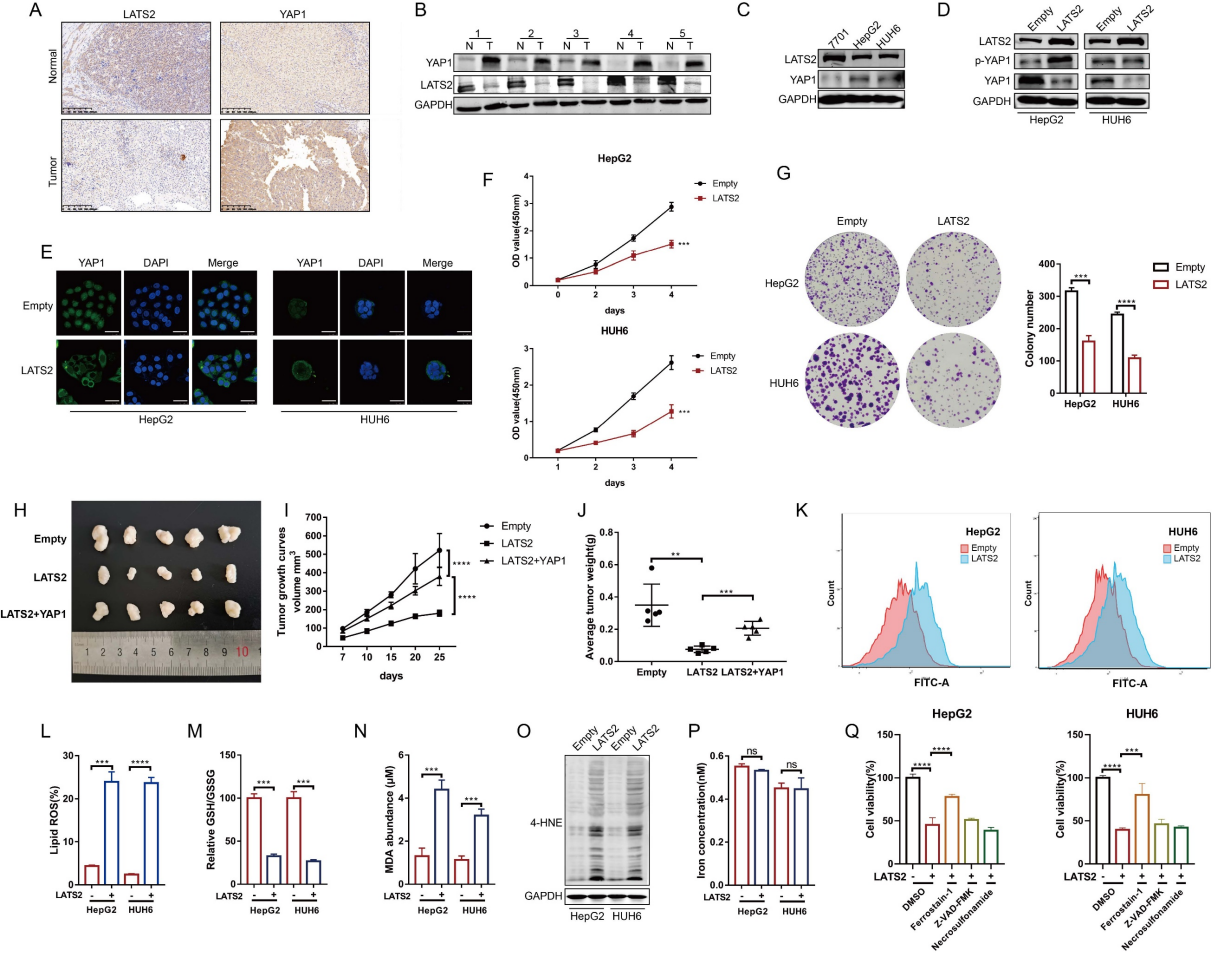
** Decreased expression of LATS2 facilitates HB proliferation by inhibiting ferroptosis.** (A and B) The protein expression of LATS2 and YAP1 in HB tissues and adjacent tissues was determined by IHC (A) and WB (B). Scale bar: 200 μm. (C) LATS2 and YAP1 expression in HB cell lines compared with that in QSG-7701 hepatocytes was determined by WB. (D) The protein expression of LATS2 and YAP1 upon LATS2 overexpression was measured by WB. (E) Localization of YAP1 in HB cell lines was measured by confocal microscopy. Scale bar, 50 μm. (F and G) The role of LATS2 in the proliferation of HBs was evaluated by CCK8 (F) and colony formation (G) assays. (H) A xenograft mouse model was generated under the indicated conditions using HepG2 cell lines (n = 5). (I) Tumor volumes were measured and calculated on the indicated days using the following formula: Volume (mm^3^) = Length (mm) × Width^2^ (mm^2^)/2. (J) The weights of the resected tumors were measured. (K and L) Lipid ROS levels were measured BY C11-Bodipy staining coupled with flow cytometry. (M-O) Ferroptotic events were evaluated in cells with LATS2 overexpression, including the GSH/GSSG ratio (M) and MDA production (N) and 4-HNE level (O). (P) Iron concentration was detected in cells with LATS2 overexpression. (Q) CCK8 assays were conducted with LATS2 overexpression in the presence of DMSO, ferrostatin-1 (1 μM), Z-VAD-FMK (10 μM), or necrosulfonamide (1 μM). All quantitative data are shown as the mean ± SD from three independent experiments. **p <0.01, ***p <0.001, ****p <0.0001; scale bars, 200 μm.

Previous studies investigating the role of the Hippo pathway in HB tumorigenesis have focused mainly on the YAP1 protein. However, the underlying regulatory mechanism of LATS2, a core kinase in the Hippo pathway, has not yet been fully illustrated. To investigate the expression of LATS2 in HB, we conducted immunohistochemistry (IHC) assays in HB tissues and paired adjacent normal tissues. The results indicated decreased expression of LATS2 and increased expression of YAP1 in HB tissues (Figure 1A). In addition, western blotting (WB) assays validated the expression of LATS2 and YAP1 in five paired HB tissues (Figure 1B) and in the HB cell lines HepG2 and HUH6 (Figure 1C). Considering the decreased expression of LATS2 in HB, we overexpressed LATS2 to investigate its biological role in HB (Figure 1D). Overexpression of LATS2 significantly increased YAP1 phosphorylation, thereby inhibiting YAP1 nuclear localization, as expected (Figure 1D and E). CCK8 and colony formation assays indicated that increased LATS2 considerably inhibited the proliferation of the HB cell lines (Figure 1F and G). In addition, the inhibition of tumorigenesis in a xenograft mouse model showed similar results when LATS2 was overexpressed, and this effect could be reversed by the simultaneous overexpression of YAP1 (Figure 1H-J). Furthermore, we conducted a flow cytometry assay to evaluate the level of apoptosis. We found that the degree of apoptosis only partially explained the inhibition of cell proliferation caused by LATS2 overexpression Supplementary Figure 1A and B), indicating that other types of programmed cell death are involved. The Hippo pathway has recently been reported to regulate ferroptosis; however, its potential role in tumorigenesis remains controversial (16, 21. Intriguingly, we observed an increase in the accumulation of lipid ROS when LATS2 was overexpressed, as demonstrated by C11-BODIPY staining (Figure 1K and L). The GSH/GSSG ratio, which represents cellular oxidative stress, was significantly decreased upon LATS2 overexpression (Figure 1M). Moreover, the lipid peroxidation products malondialdehyde (MDA) and 4-hydroxynonenal (4-HNE) were elevated (Figure 1N and O), whereas the concentration of iron remained unaffected (Figure 1P). Moreover, the suppression of cell proliferation mediated by LATS2 overexpression was obviously reversed by the ferroptosis inhibitor ferrostatin-1 and slightly reversed by the apoptosis inhibitor ZVAD-FMK. By contrast, treatment with the necroptosis inhibitor necrosulfonamide did not affect HepG2 or HUH6 cells (Figure 1Q).

Taken together, the results confirmed the decreased expression of LATS2 in HB and that overexpression of LATS2 could induce ferroptosis by affecting GSH generation.

### 3.2 LATS2 serves as a potential diagnostic and prognostic biomarker for HB patients

**Figure 2:**
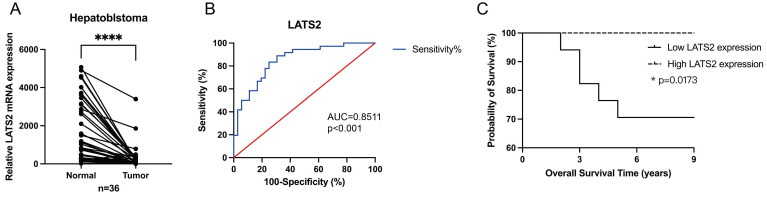
** LATS2 serves as a potential diagnostic and prognostic biomarker for HB patients.** (A) Relative expression of LATS2 in 36 pairs of HB tissues and matched normal tissues, determined by qRT‒PCR. (B) The ROC curve was used to measure the diagnostic value of LATS2. (C) Kaplan‒Meier analyses revealed correlations between LATS2 expression levels and the OS of HB patients. All quantitative data are shown as the mean ± SD from three independent experiments. *p <0.05, ****p <0.0001.

Next, we investigated the potential role of LATS2 as a biomarker candidate in HB. The expression of LATS2 was further verified in 36 pairs of HB tissues and matched normal tissues by qRT‒PCR, and the results confirmed the significant increase in LATS2 expression in HB tissues compared with that in normal tissues (Figure 2A), which was consistent with the WB and IHC results. The clinicopathological characteristics of the patients included in our study are shown in Table 1. The expression levels of LATS2 were used to divide patients into high and low expression groups based on the median △△Ct value obtained by qRT‒PCR. The correlations between LATS2 expression levels and clinical features were analyzed and are displayed in Table 2. A significant correlation between LATS2 expression and PRETEXT expression was identified (p = 0.035), while no correlation was observed between LATS2 expression and other clinicopathological factors. Subsequently, we generated a receiver operating characteristic (ROC) curve to investigate the diagnostic value of LATS2. The area under the curve (AUC) was 0.8511 between tumor and normal tissues, representing a powerful diagnostic capability (Figure 2B). Kaplan-Meier curves were used to evaluate the prognostic role of LATS2 in patients with HB, and the results indicated that low expression of LATS2 predicted worse survival (Figure 2C). These data suggest that LATS2 might be a promising biomarker and potential therapeutic target for HB patients.

### 3.3 LATS2 mediates the reduction of GSH by targeting PSAT1 in HB cells

**Figure 3:**
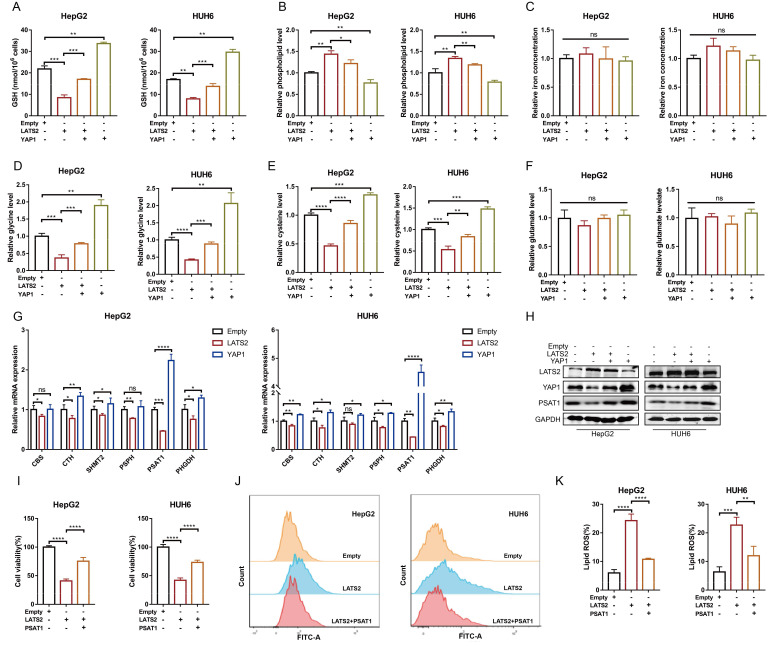
** LATS2 mediates the reduction in GSH levels by targeting PSAT1 in HB cells.** (A-C) HepG2 and HUH6 cells were transfected with LATS2. YAP1 was simultaneously overexpressed, and ferroptosis-related metabolic parameters, including the GSH concentration (A), phospholipid level (B), and iron concentration (C), were measured. (D-F) The relative levels of glycine (D), cysteine (E), and glutamine were measured under the indicated conditions. (G) Transcriptional levels of the key enzymes of the serine synthesis pathway were screened by qRT‒PCR after LATS2 overexpression, with or without YAP1 overexpression. (H) The expression of LATS2, YAP1, and PSAT1 was measured by WB under the indicated conditions. (I) CCK-8 assays were conducted after LATS2 overexpression, with or without PSAT1 overexpression. (J and K) Lipid ROS levels were measured by C11-Bodipy staining coupled with flow cytometry under the indicated treatments. All quantitative data are shown as the mean ± SD from three independent experiments. n.s., no significant difference, *p <0.05, **p <0.01, ***p <0.001, ****p <0.0001.

The initiation of ferroptosis involves GSH, lipid, and iron metabolism. To investigate the underlying pathways regulated by LATS2, we detected relevant metabolites associated with LATS2 overexpression. LATS2 overexpression significantly decreased the GSH concentration and increased the phospholipid concentration but did not affect the iron concentration (Figure 3A-C). In addition, these altered metabolites were simultaneously reversed by YAP1 overexpression (Figure 3A-C). Because the production of GSH can be stimulated by cysteine, glycine, and glutamate, we further evaluated the levels of these metabolites. LATS2 overexpression decreased the concentrations of glycine and cysteine without influencing that of glutamate (Figure 3D-F). Correspondingly, simultaneous overexpression of YAP1 reversed the changes in glycine and cysteine concentrations (Figure 3D-F). Since both glycine and cysteine are produced by the serine synthesis pathway (SSP) 22, 23 Supplementary Figure 2A), we further investigated whether the Hippo pathway regulates glycine and cysteine via this metabolic axis. The transcription levels of enzymes in the SSP were measured, and the results indicated that overexpression of LATS2 or YAP1 influenced most of the enzymes involved, especially phosphoserine aminotransferase 1 (PSAT1) (Figure 3G). We speculated that PSAT1 might be a target of the Hippo pathway. We found that overexpression of LATS2 directly inhibited PSAT1 expression, while YAP1 increased PSAT1 expression (Figure 3H). Moreover, the protein stability of PSAT1 was not obviously altered (Supplementary Figure 2B), suggesting that the Hippo pathway might affect its transcription level. Subsequently, we found that the inhibition of cell proliferation mediated by increased LATS2 expression was largely reversed by PSAT1 overexpression in HepG2 and HUH6 cells (Figure 3I). Moreover, LATS2-mediated lipid ROS accumulation was inhibited by PSAT1 overexpression (Figure 3J and K). Similarly, PSAT1 reversed the changes in the levels of the corresponding metabolites, including MDA, glycine, and cysteine, mediated by LATS2 (Supplementary Figure 2D and E). Taken together, these results suggest that LATS2 reduces GSH levels by targeting PSAT1 in HB cells.

### 3.4 LATS2 stimulates PSAT1 transcription via the YAP1/ATF4 pathway

**Figure 4:**
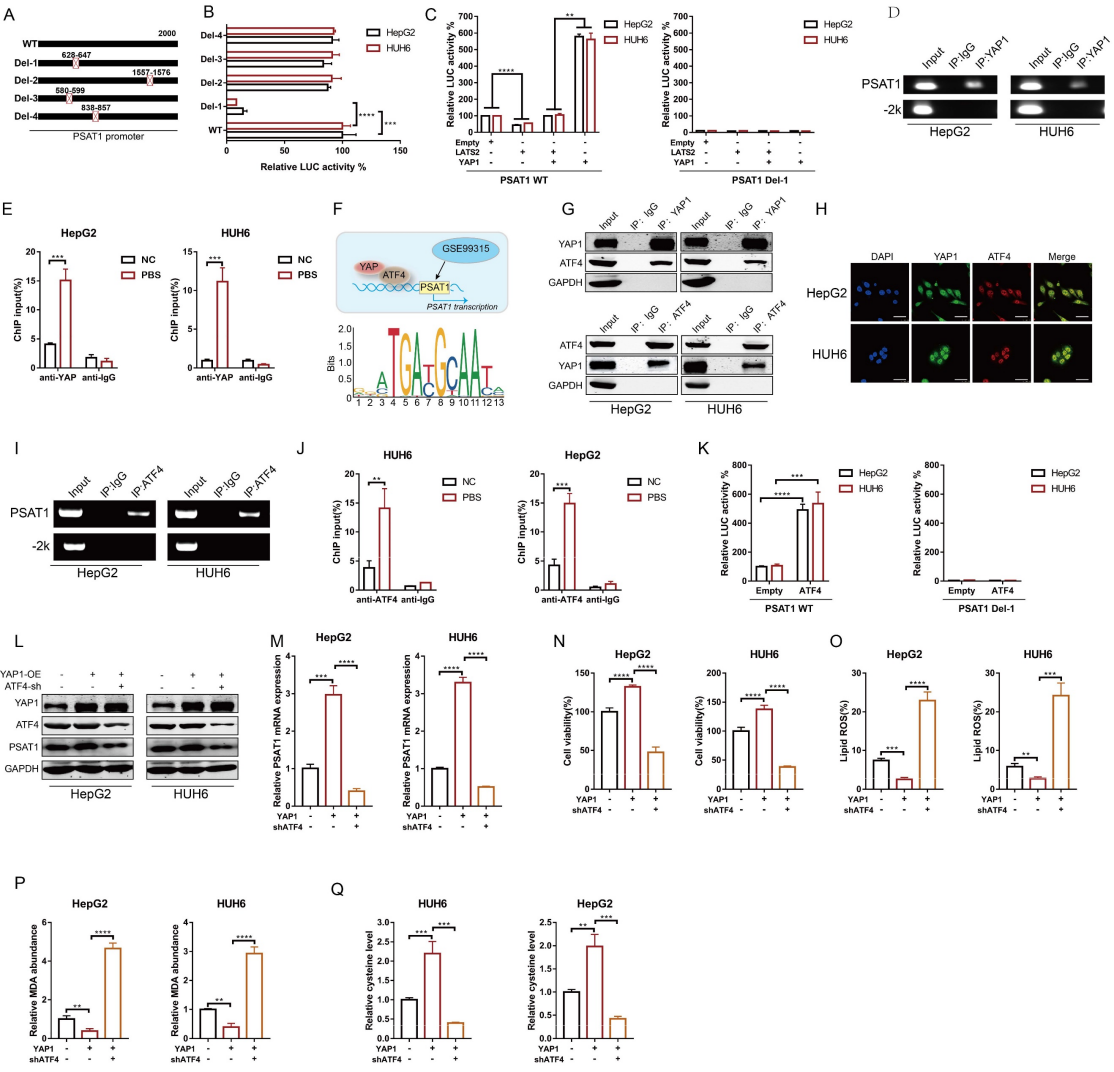
** LATS2 stimulates PSAT1 transcription via the YAP/ATF4 pathway.** (A and B) The promoter of PSAT1 was mutated as indicated (A), and luciferase activity was measured using a dual-luciferase system after the indicated vectors were transfected into HepG2 and HUH6 cells (B). (C) The PSAT1 promoter activity of wild-type (WT) and mutant (Del-1) strains was measured using a dual-luciferase system in HepG2 and HUH6 cells with simultaneous overexpression of LATS2, with or without YAP1. (D and E) Chromatin was immunoprecipitated using an anti-YAP1 antibody or negative control anti-IgG antibody (D), and qRT-PCR (E) was then conducted on HepG2 and HUH6 cells. (F) Predicted binding site (PBS) of YAP1 and the promoter of PSAT1 according to bioinformatics analysis of GSE99315. (G) Reciprocal co-IP of YAP1 and ATF4 was performed using anti-YAP1 and anti-ATF4 antibodies, respectively, followed by WB using the indicated antibodies in HepG2 and HUH6 cells. (H) Colocalization of YAP1 and ATF4 in HepG2 and HUH6 cells, as measured by confocal microscopy. Scale bar, 50 μm. (I and J) Chromatin was immunoprecipitated using an anti-ATF4 antibody or negative control anti-IgG antibody (I), and qPCR (J) was then conducted in HepG2 and HUH6 cells. (K) The PSAT1 promoter activity of the WT and Del-1 strains was measured using a dual-luciferase system in HepG2 and HUH6 cells with simultaneous overexpression of PSAT1. (L and M) The protein (L) and mRNA (M) expression levels of PSAT1 were measured under the indicated treatments. (N-P) Ferroptotic events in YAP1-overexpressing cells with or without ATF4 knockdown were evaluated by measuring cell viability (N), lipid ROS levels (O), and MDA production (P). (Q) The relative level of cysteine was detected under the indicated conditions. All quantitative data are shown as the mean ± SD from three independent experiments. **p <0.01, ***p <0.001, ****p <0.0001.

Since LATS2 induces PSAT1 expression by affecting its transcription, we next investigated the potential underlying mechanisms involved. YAP1, the main downstream effector of LATS2, functions as a transcriptional cofactor. We conducted a bioinformatics analysis using the ChIP-seq dataset GSE99315 and confirmed multiple potential YAP1 binding sites in the PSAT1 promoter region (Supplementary Table 1. Accordingly, we generated the indicated reporters containing PSAT1 promoter mutants named Del-1 to Del-4 (Figure 4A). Deletion of the -647 to -628 nt region (referred to as Del-1, the highest ranking of predicted binding sites) decreased the basal promoter activity of PSAT1 to a level similar to that of the pGL3 control vector (Figure 4B), suggesting that this region might be responsible for YAP1 binding. To confirm the regulation of PSAT1 promoter activity by LATS2 and YAP1, we overexpressed LATS2 with or without YAP1 in HepG2 and HUH6 cells (Figure 4C). As expected, overexpression of LATS2 inhibited PSAT1 promoter activity, whereas overexpression of YAP1 significantly increased PSAT1 promoter activity (Figure 4C). Importantly, changes in LATS2/YAP1 expression did not affect the activity of the PSAT1 promoter with deletion of the -647 to -628 nt region (Del-1) (Figure 4C). Furthermore, physical binding between YAP1 and the PSAT1 promoter was confirmed using a ChIP assay (Figure 4D and E).

YAP/TAZ lack DNA-binding domains, thus relying on other transcription factors to mediate their transcriptional output. It has been reported that activating transcription factor 4 (ATF4) transcriptionally regulates PSAT1 expression (Figure 4F). Thus, we investigated whether YAP1 regulated PSAT1 expression via ATF4. Co-IP assays were conducted to validate the interaction between YAP1 and ATF4. Endogenous ATF4 was observed in the immunoprecipitates pulled down by the anti-YAP1 antibody, and vice versa (Figure 4G). Furthermore, colocalization of YAP1 and ATF4 was confirmed in HepG2 and HUH6 cells (Figure 4H). Moreover, physical binding between ATF4 and the PSAT1 promoter was confirmed using a ChIP assay (Figure 4I and J). Overexpression of ATF4 significantly increased PSAT1 promoter activity but had no effect on the activity of the PSAT1 promoter with Del-1 (Figure 4K). To confirm the potential role of ATF4 in YAP1-mediated regulation of PSAT1 expression, we overexpressed YAP1 in the presence or absence of simultaneous ATF4 knockdown (Figure 4L). The results indicated that PSAT1 expression was obviously increased by YAP1 overexpression and that this effect was abolished by ATF4 knockdown (Figure 4L and M). Moreover, simultaneous ATF4 knockdown increased YAP1-mediated inhibition of ferroptosis, as assessed by cell viability assays (Figure 4N), along with the production of lipid ROS (Figure 4O), MDA (Figure 4P), and glycine (Figure 4Q).

### 3.5 METTL3 mediates LATS2 mRNA instability and expression via m6A modification

**Figure 5:**
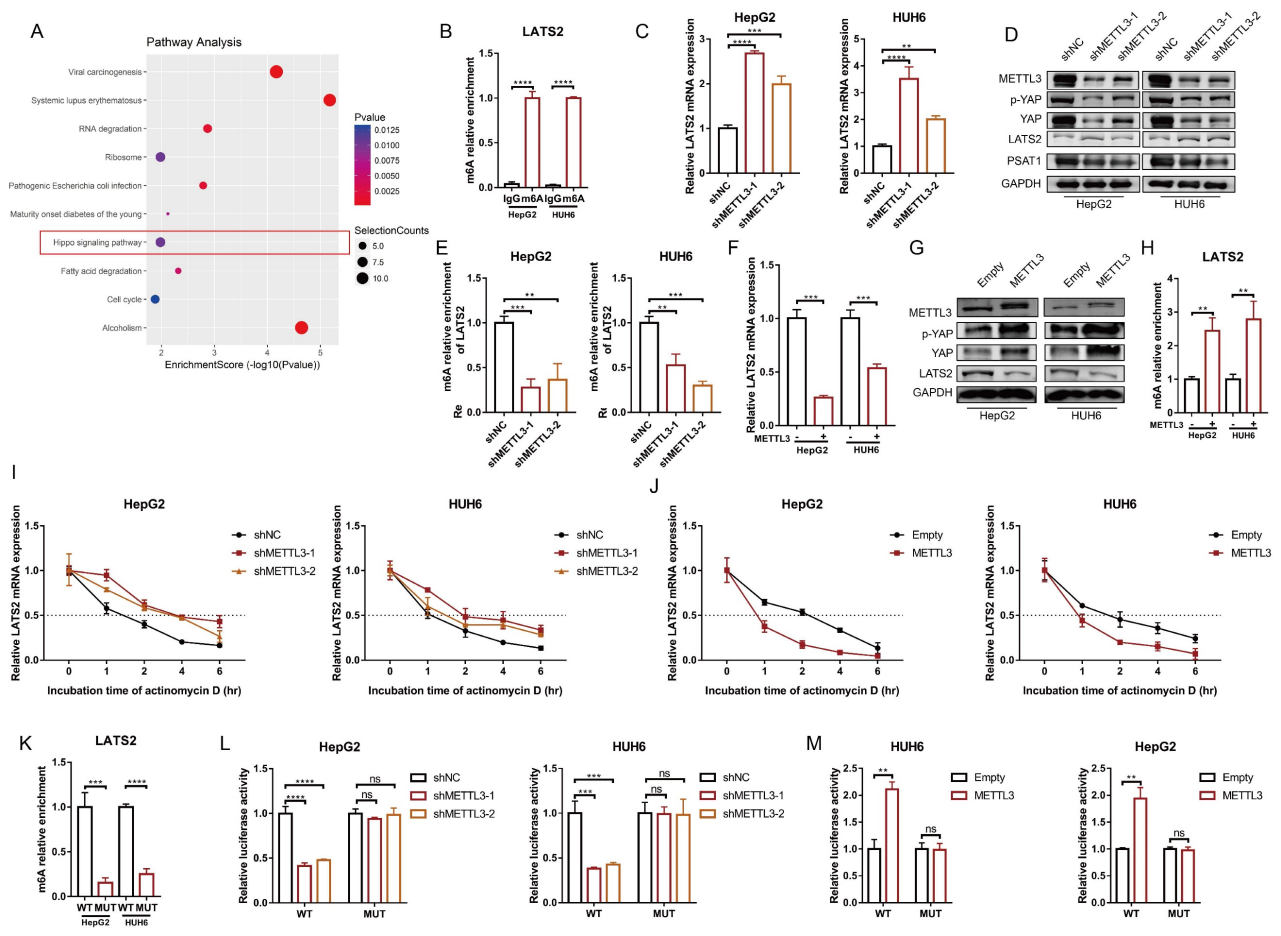
** METTL3 mediates LATS2 mRNA instability and expression via m6A modification.** (A) Corresponding KEGG pathways were identified according to the significant genes with upregulated m6A methylation. (B) The relative m6A enrichment levels of the LATS2 mRNA 5′UTR were verified via meRIP-qPCR analysis in HepG2 and HUH6 cells. (C-E) The mRNA expression of LATS2 (C), the protein expression of METTL3, YAP1, p-YAP1, and LATS2 (D), and the relative m6A enrichment of LATS2 (E) were measured with or without METTL3 knockdown. (F-H) The mRNA expression of LATS2 (F), the protein expression of METTL3, YAP1, p-YAP1, and LATS2 (G), and the relative m6A enrichment of LATS2 (H) were measured in cells with or without METTL3 overexpression. (I and J) The decay rate of LATS2 mRNA was detected via qRT-PCR at the indicated time points after METTL3 knockdown (I) or overexpression (J) in HUH6 and HepG2 cells treated with ActD (5 μg/ml). (K) The m6A levels of the WT and Mut pmir-GlO plasmids were verified by MeRIP-qPCR. (L and M) Luciferase activities of the WT and Mut pmir-GlO plasmids were measured after METTL3 knockdown (L) or overexpression (M) in HepG2 and HUH6 cells. All quantitative data are presented as the mean ± SD from three independent experiments. WT, wild-type; Mut, mutation; n.s., no significant difference; **p <0.01, ***p <0.001, ****p <0.0001.

Next, we investigated the underlying mechanism of decreased LATS2 expression in HB. Our previous study generated a transcriptome-wide m6A map and demonstrated that upregulation of m6A modification facilitates HB proliferation 11. The significant genes with upregulated m6A methylation were selected, and the corresponding KEGG pathways, including the Hippo signaling pathway, were identified (Figure 5A). Notably, the m6A modification of LATS2 mRNA was abnormally increased in HB tissues (Supplementary Excel 1). MeRIP-qPCR was conducted to validate the m6A methylation of LATS2 mRNA in HepG2 and HUH6 cells (Figure 5B). METTL3 is the main enzyme that initiates m6A modification, suggesting that METTL3 might regulate the m6A methylation of LATS2 mRNA. To verify our hypothesis, we knocked down METTL3 in HepG2 and HUH6 cells Supplementary Figure 3B), and the results indicated that downregulation of METTL3 significantly increased the levels of LATS2 mRNA and protein, subsequently leading to a decrease in YAP1 expression (Figure 5C and D). Consistent with the role of PSAT1 as a downstream target of the LATS2/YAP1 pathway, we also detected a decrease in PSAT1 protein levels in the samples in which METTL3 was downregulated (Figure 5D). These additional data support our conclusion that METTL3 plays a crucial role in regulating the LATS2/YAP1 signaling pathway, with downstream effects on PSAT1 expression. Correspondingly, a significant reduction in LATS2 expression was observed when METTL3 was overexpressed, accompanied by increased expression of YAP1 (Figure 5F, Supplementary Figure 3C). Furthermore, MeRIP-qPCR assays indicated that LATS2 mRNA could be enriched with an anti-m6A antibody and that m6A modification near the putative m6A site was significantly reduced after METTL3 knockdown (Figure 5E), which subsequently enhanced the mRNA stability of LATS2 (Figure 5I). By contrast, METTL3 overexpression significantly inhibited LATS2 expression (Figure 5F) and correspondingly increased YAP1 expression (Figure 5G). Moreover, METTL3 overexpression increased LATS2 m6A modification (Figure 5H) but decreased mRNA stability (Figure 5J). According to our previous data (Supplementary Figure 3A), m6A modification sites are located in the 5' untranslated region (5'UTR) of mRNAs. Thus, bioinformatics analyses were conducted to predict the putative m6A site using RMBase v2.0 (http://rna.sysu.edu.cn/rmbase/) (Supplementary Figure 3D). To determine whether METTL3 destabilizes LATS2 mRNA via the putative m6A site, we cloned the WT 5'UTR and MUT 5'UTR (GGAC to GGCC) of LATS2 downstream of the firefly luciferase-encoding region in the pmir-GLO vector (Supplementary Figure 3E). The MeRIP-qPCR results indicated that the WT 5'UTR of LATS2 was highly enriched compared with the MUT 5'UTR (Figure 5K). Moreover, luciferase activity was largely reduced when METTL3 was knocked down and was significantly increased with METTL3 overexpression (Figure 5L and M). However, these effects were abolished when the MUT reporter was introduced (Figure 5L and M). Taken together, these results indicate that METTL3 mediates the m6A methylation of LATS2 mRNA and regulates its mRNA stability.

### 3.6 YTHDF2 recognizes the m6A modification of LATS2 mRNA and mediates its decay

**Figure 6:**
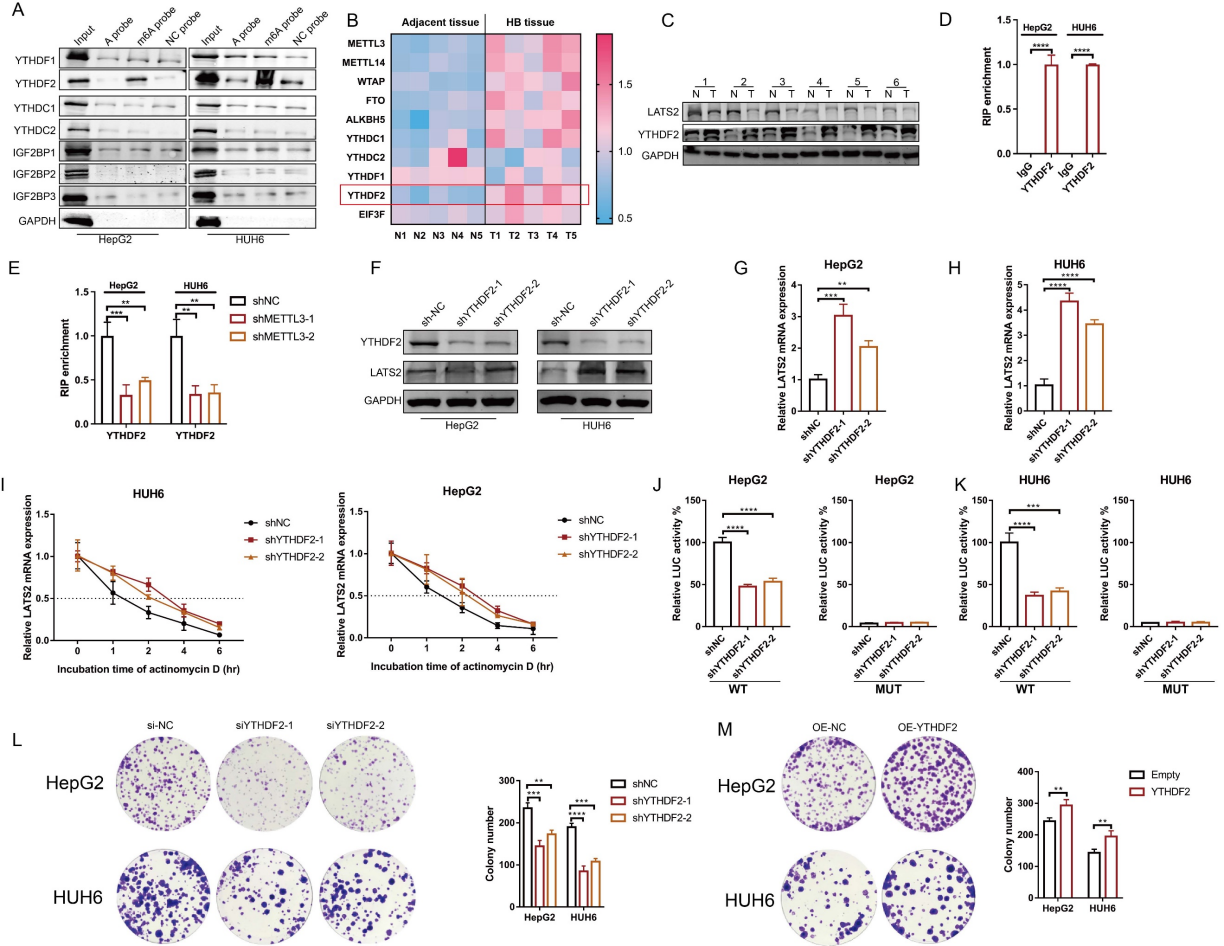
** YTHDF2 recognizes the m6A modification of LATS2 mRNA and mediates its decay.** (A) RNA pulldown followed by WB was conducted to identify putative m6A readers in HepG2 and HUH6 cell lysates incubated with synthetic LATS2 probes (A probe, m6A probe, and UTR-NC probe). (B) Heatmap analysis of m6A methylation-related proteins based on proteomic data of five paired HB and normal tissues. (C) The protein expression of LATS2 and YTHDF2 in HB tissues and adjacent tissues was determined by WB. (D) RIP-qPCR results demonstrating the association of the LATS2 5′UTR with YTHDF2 in HepG2 and HUH6 cells. (E) RIP-qPCR was conducted to confirm the binding of YTHDF2 to the LATS2 5′UTR in HepG2 and HUH6 cells with METTL3 knockdown. (F-H) The protein expression of LATS2 (F) and mRNA expression of LATS2 (G and H) were measured with or without YTHDF2 knockdown. (I) The half-life of the LATS2 mRNA was measured via qRT-PCR at the indicated time points after YTHDF2 knockdown. (J and K) Luciferase activities of the WT and Mut pmir-GlO plasmids were measured after YTHDF2 knockdown in HepG2 (J) and HUH6 (K) cells. (L and M) Colony formation assays were conducted to evaluate the role of YTHDF2 in the proliferation of HepG2 and HUH6 cells in which YTHDF2 is knocked down (L) or overexpressed (M). All quantitative data are shown as the mean ± SD from three independent experiments. **p <0.01, ***p <0.001, ****p <0.0001.

m6A readers determine mRNA fate by recognizing m6A modifications. To identify m6A readers that recognize the m6A modification of LATS2, we synthesized partial LATS2 5'UTR probes (Supplementary Figure 4A) and conducted an RNA pulldown assay. The results indicated that YTHDF2 was pulled down by the LATS2 5'UTR probe with m6A modification at the GGAC motif but not by the probe without m6A modification (Figure 6A). Our previous proteomic analysis of five paired HB and normal tissues revealed that YTHDF2 expression is dramatically increased in tumor tissues (Figure 6B, Supplementary Excel 3). In addition, the abundance of METTL3 mRNA increased, which is consistent with previous results. Subsequently, we verified YTHDF2 expression in HB tissues by WB. The results confirmed the elevated expression of YTHDF2 (Figure 6C). These data indicate that YTHDF2 might be the m6A modification reader that recognizes LATS2 mRNA. Then, RIP-qPCR was conducted to confirm the physical interaction between YTHDF2 and the 5'UTR of LATS2 mRNA in the HepG2 and HUH6 cell lines (Figure 6D). This interaction decreased after METTL3 knockdown, indicating that m6A modification regulates the physical binding of LATS2 mRNA and YTHDF2 (Figure 6E). Next, we knocked down YTHDF2 and found that the protein and mRNA expression levels of LATS2 were dramatically increased (Figure 6F-H). Correspondingly, the overexpression of YTHDF2 led to decreased expression of LATS2 (Supplementary Figure 4B and C). Moreover, the stability of the LATS2 mRNA was significantly enhanced by YTHDF2 downregulation (Figure 6I). Notably, the luciferase activity of the LATS2 WT reporter was significantly reduced when YTHDF2 was knocked down compared with that of the MUT reporter, indicating that YTHDF2 interacts with LATS2 mRNA in a m6A-dependent manner. These data demonstrate that YTHDF2 recognizes m6A-methylated LATS2 mRNA and mediates its decay. In addition, we assessed the biological function of YTHDF2 in HB tumorigenesis using CCK8 and colony formation assays. The results showed a significant reduction in cell viability and colony formation capacity following the knockdown of YTHDF2 in the HepG2 and HUH6 cell lines. By contrast, YTHDF2 overexpression increased cell proliferation and colony formation (Figure 6L and M, Supplementary Figure 4D and E).

### 3.7 YTHDF2 facilitates LATS2 mRNA deadenylation by recruiting the CCR4-NOT complex

**Figure 7:**
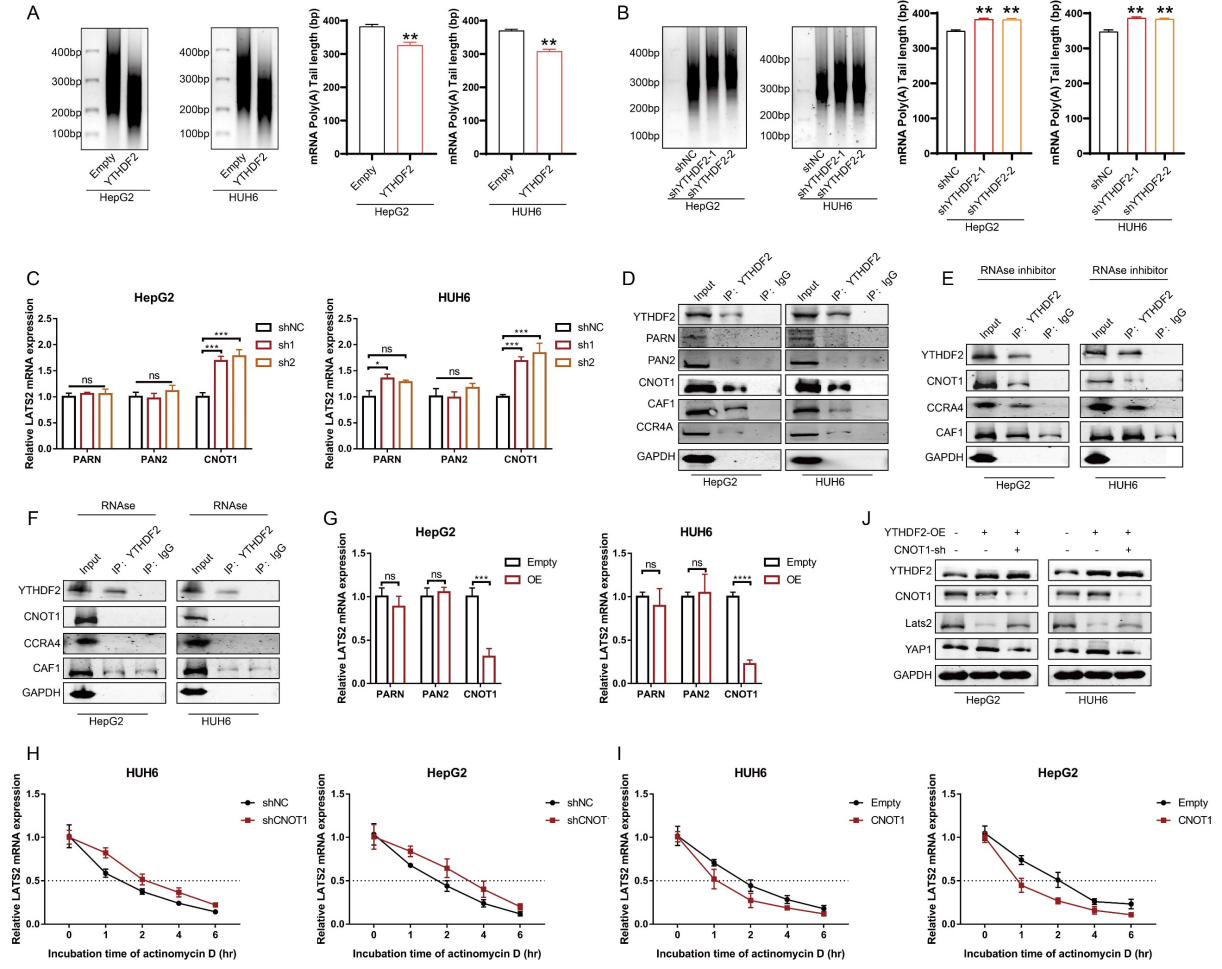
** YTHDF2 facilitates LATS2 mRNA deadenylation by recruiting the CCR4-NOT complex.** (A and B) The poly(A) tail length of the endogenous LATS2 transcript after YTHDF2 overexpression (A) or knockdown (B) was detected via RACE-PAT assays in HUH6 and HepG2 cells. (C) The transcriptional level of LATS2 was detected using qRT-PCR to confirm the presence of deadenylation-related enzymes in HepG2 and HUH6 cells. (D) Coimmunoprecipitation of YTHDF2 with the indicated enzymes in HepG2 and HUH6 cells. (E and F) Coimmunoprecipitation of YTHDF2 with the indicated enzymes in HepG2 and HUH6 cells treated with RNase inhibitor (E) or RNase (F). (G) The expression of LATS2 mRNA was measured using qRT-PCR following the overexpression of the indicated enzymes. (H and I) The decay rate of LATS2 mRNA was detected via qRT-PCR at the indicated time points after CNOT1 knockdown (H) or overexpression (I) in HepG2 and HUH6 cells. (J) The protein levels of LATS2 and YAP1 were measured in cells with spontaneous CNOT1 knockdown and in which YTHDF2 was overexpressed. All quantitative data are shown as the mean ± SD from three independent experiments. n.s., no significant difference, *p <0.05, **p <0.01, ***p <0.001, ****p <0.0001.

It has been reported that the binding of YTHDF2 results in alterations in the translation efficiency and stability of m6A-containing RNAs through accelerated deadenylation (24. Thus, we conducted a RACE-PAT assay to measure the poly(A) tail length of the LATS2 mRNA. The length of the poly(A) tail of the LATS2 mRNA decreased after YTHDF2 overexpression and increased after YTHDF2 knockdown (Figure 7A and B). mRNA deadenylation is triggered by deadenylases, including PARN, the PAN2-PAN3 complex, and the CCR4-NOT complex. We knocked down the relevant enzymes to investigate whether the YTHDF2-mediated increase in LATS2 mRNA expression is related to deadenylation, as indicated in Figure 7C. The results indicated that inhibition of CNOT1, a large scaffold subunit of the CCR4-NOT complex, significantly increased LATS2 mRNA expression. However, this effect was not observed upon inhibition of PARN and PAN2. Furthermore, we conducted co-IP assays to explore the physical binding between YTHDF2 and deadenylases. The results indicated that YTHDF2 directly binds to CNOT, CAF1, and CCR4A but not to PARN or PAN2 (Figure 7D). These results demonstrate that the CCR4-NOT complex mediates LATS2 mRNA deadenylation. Next, we treated HepG2 and HUH6 cells with an RNase inhibitor or RNase A and conducted co-IP assays. Co-IP of endogenous CNOT, CAF1, and CCR4A was observed in the immunoprecipitates pulled down by the anti-YTHDF2 antibody in cells treated with the RNase inhibitor but not in those treated with RNase A (Figure 7E and F), indicating that physical binding between YTHDF2 and the CCR4-NOT complex relies on the presence of RNA. Correspondingly, we overexpressed CNOT1, PARN, and PAN2 in HepG2 and HUH6 cells to further verify the regulatory effect of deadenylases on LATS2 mRNA expression. The results indicated that CNOT1 overexpression significantly decreased LATS2 mRNA expression (Figure 7G). Moreover, LATS2 mRNA stability was negatively related to CNOT1 expression (Figure 7H and I). In addition, YTHDF2 overexpression decreased the expression of LATS2 and subsequently led to increased YAP1 expression (Figure 7J). This phenomenon was abolished when CNOT1 was simultaneously knocked down (Figure 7J). Taken together, these results indicate that YTHDF2 mediates the instability and expression of LATS2 mRNA by recruiting the CCR4-NOT complex, thereby enhancing its mRNA deadenylation.

## 4. Discussion

**Figure 8:**
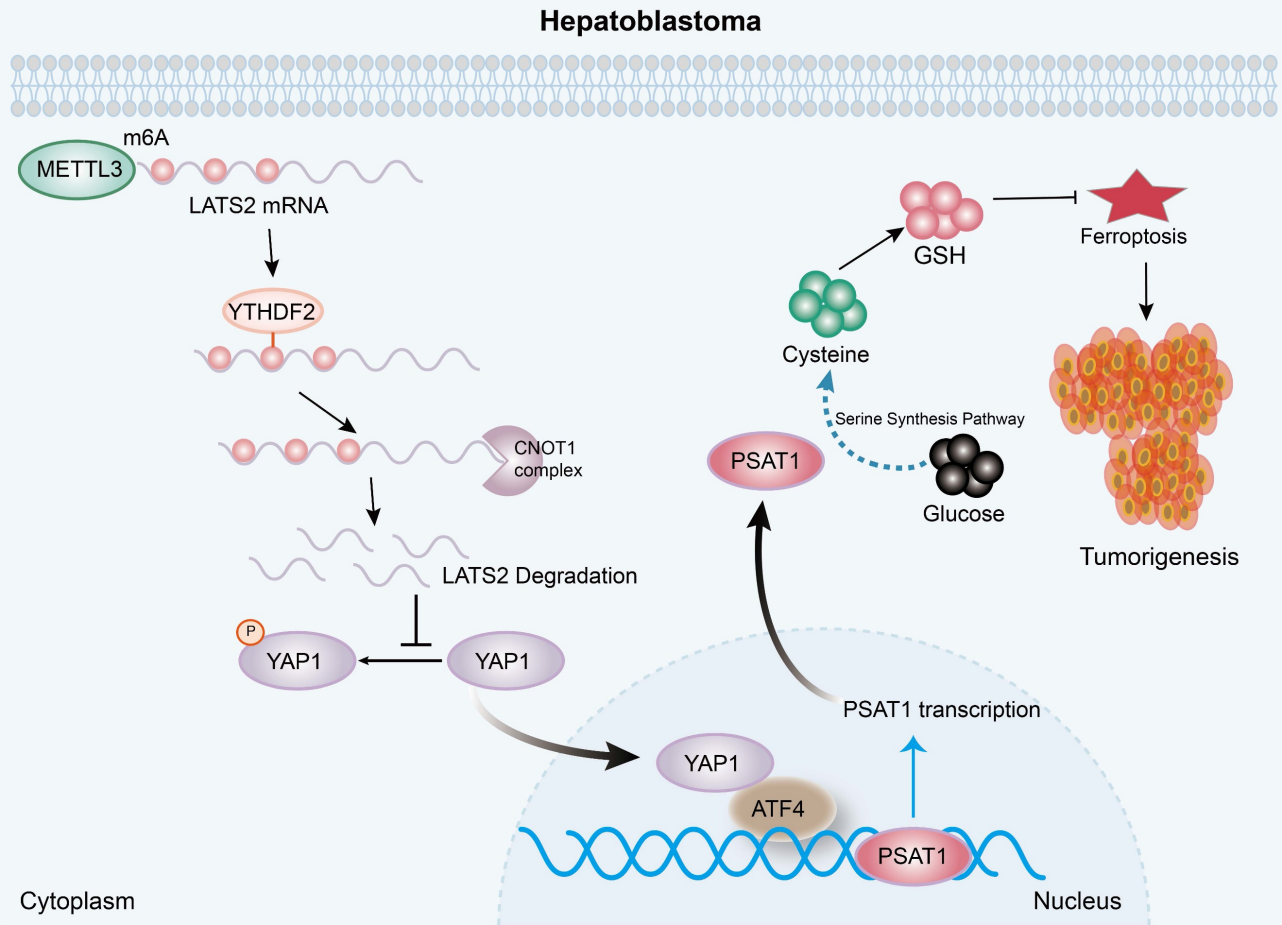
** A diagram illustrating the underlying molecular mechanism identified in the present study.** Mechanistically, increased m6A modification of the LATS2 mRNA, which is recognized by YTHDF2, facilitates its degradation through the CCR4-NOT complex. Reduced LATS2 expression activates YAP1, promoting GSH synthesis via the ATF4/PSAT1 pathway, ultimately enhancing ferroptosis resistance.

In recent decades, numerous studies have confirmed the deregulation of the Hippo pathway and the activation of its downstream effector, YAP1, in multiple hepatic malignancies, including HB. It has been reported that YAP1 is activated in approximately 70-80% of HB specimens and is associated with poor prognosis 20, 25. LATS2, the core component of the Hippo cascade, is a well-known tumor suppressor protein. The expression levels of LATS2 are significantly reduced in various types of cancer, such as liver, gastric, and colorectal cancer, resulting in an enhanced antiapoptotic ability of tumor cells 26, 27. However, the role of LATS2 in HB has not yet been investigated. In this study, we revealed decreased LATS2 expression regulated by m6A modification. Decreased LATS2 expression activates the Hippo pathway and enhances the ferroptosis resistance of HB (Figure 8).

Ferroptosis is a recently discovered form of programmed cell death characterized by the iron-dependent accumulation of peroxidated phospholipids 28. The classical regulatory mechanism of ferroptosis consists of iron, amino acid, and lipid metabolism. Since the first study published in *Nature* by Jiang's laboratory reporting that YAP1 regulates cancer cell ferroptosis via acyl-CoA synthetase long-chain family member 4 (ACSL4) and the transferrin receptor^16^, the regulatory role of YAP1 in ferroptosis has attracted significant attention from researchers. Yang et al. also showed that YAP1 overexpression sensitizes cancer cells to ferroptosis via SKP2 29. However, the results of another study suggested that YAP1 inhibits ferroptosis by reducing ferritinophagy in hepatocytes 21. Thus, the comprehensive role of YAP1 in ferroptosis initiation remains controversial. In this study, we found that decreased LATS2-mediated YAP1 activation confers ferroptosis resistance by facilitating the synthesis of GSH, which is the most abundant endogenous antioxidant and is composed of glycine, cysteine, and glutamate 30.

The SSP plays a pivotal role in redox homeostasis by producing L-serine, the metabolic precursor of cysteine and glycine, thereby leading to GSH biosynthesis 31, 32. PSAT1 catalyzes the intermediate step within the three steps of SPP, which is the pyridoxal 5'-phosphate-dependent transamination of phosphohydroxypyruvate (pPYR) to phosphoserine (pSER) 33. PSAT1 overexpression has been reported to facilitate cancer progression in multiple studies 34, 35. In this study, we evaluated the transcription of the main enzymes associated with SPP and found that the overexpression of LATS2 or YAP1 most strongly influenced PSAT1. Similarly, Yang et al. confirmed that YAP1 promotes the expression of PSAT1 and that there is a strong positive correlation between YAP1 activity and PSAT1 expression; however, the underlying mechanism is not known 36. By conducting bioinformatics analysis using ChIP-seq datasets, we confirmed the physical binding between YAP1 and the promoter of PSAT1. Furthermore, this physical binding relies on the transcription factor ATF4. Consistent with our results, Gao et al. confirmed that PSAT1 is a target of ATF4 in breast cancer 37. However, the interaction between YAP1 and ATF4 has never been reported. This study is the first to report that YAP1 facilitates GSH synthesis via the ATF4/PSAT1 pathway.

As the core kinase of the Hippo signaling pathway, LATS2 regulates cancer progression by affecting YAP1 activity 38. YAP1 activation, accompanied by decreased LATS2 expression, has been reported in various cancers 39. However, the underlying mechanism of decreased LATS2 expression remains largely unknown. Here, we first reported that METTL3 mediates the m6A modification of LATS2 mRNA, and an RNA pulldown assay indicated that this modification is recognized by YTHDF2. Furthermore, we found that YTHDF2 recognition destabilized LATS2 mRNA and led to mRNA degradation in an m6A-dependent manner. The degradation of mRNA in eukaryotes relies on the removal of poly(A) tails, a process known as deadenylation 40. A recent study revealed that YTHDF2 destabilizes m6A-containing RNA by directly recruiting the CCR4-NOT deadenylase complex 24. Our results also demonstrated that the CCR4-NOT complex, which YTHDF2 recruits, mediates LATS2 mRNA deadenylation.

In conclusion, the present study illustrated the oncogenic role of decreased LATS2 in HB tumorigenesis through the inhibition of ferroptosis. Mechanistically, increased m6A modification of LATS2 mRNA, which is recognized by YTHDF2, facilitates its degradation through the CCR4-NOT complex. Reduced LATS2 expression activates YAP1, promoting GSH synthesis via the ATF4/PSAT1 pathway, ultimately enhancing ferroptosis resistance.

## Supplementary Material

Supplementary figures and table.

Supplementary excel file 1: MeRIP-seq-LATS2_T v N.

Supplementary excel file 2.

Supplementary excel file 3.

## Figures and Tables

**Table 1:** Clinicopathological characteristics of HB patient samples.

Variable	n
Sex	21/15
Male/Female	
Age at Diagnosis (month)	11/25
≥24/<24	
AFP at Diagnosis (ng/ml)	28/8
≥1200/<1200	
AFP at Final Detection (ng/ml)	18/10/8
≥5/<5/NA	
PRETEXT	19/10/7
I-II/III-IV/NA	
Histology	20/11/5
MIX/E/NA	
Tumor Size (cm^3^)	19/17
≥500/<500	
Metastasis	13/23
YES/NO	

“MIX” represents mixed epithelial and mesenchymal, “E” represents epithelial, “NA” represents unknown.

**Table 2:** Statistical analysis of correlations between LATS2 expression and clinicopathological characteristics in HB patients.

HB (n=36)	PRKAA2 expression		P value
	Low	High	
Sex			>0.999
Male	10	11	
Female	8	7	
Age at Diagnosis (month)			>0.999
≥24	6	5	
<24	12	13	
AFP at Diagnosis			0.228
≥1200ng/ml	16	12	
<1200ng/ml	2	6	
AFP at Final Detection			0.628
≥5ng/ml	9	9	
<5ng/ml	4	6	
NA	5	3	
PRETEXT			0.035
I-II	6	13	
III-IV	6	4	
NA	6	1	
Histology			0.427
MIX	9	11	
E	5	6	
NA	4	1	
Tumor Size (cm^3^)			0.505
≥500	11	8	
<500	7	0	
Metastasis			0.427
YES	3	6	
NO	12	9	

## References

[1] Beyea JA, Lau C, Cooke B, Hall S, Nathan PC, Gupta S (2020). Long-Term Incidence and Predictors of Significant Hearing Loss Requiring Hearing Assistive Devices Among Childhood Cancer Survivors: A Population-Based Study. J Clin Oncol.

[2] Khanna R, Verma SK (2018). Pediatric hepatocellular carcinoma. World J Gastroenterol.

[3] Kehm RD, Osypuk TL, Poynter JN, Vock DM, Spector LG (2018). Do pregnancy characteristics contribute to rising childhood cancer incidence rates in the United States?. Pediatr Blood Cancer.

[4] Green AL, Furutani E, Ribeiro KB, Rodriguez Galindo C (2017). Death Within 1 Month of Diagnosis in Childhood Cancer: An Analysis of Risk Factors and Scope of the Problem. J Clin Oncol.

[5] Hanahan D (2022). Hallmarks of Cancer: New Dimensions. Cancer Discov.

[6] Liu T, Wei Q, Jin J, Luo Q, Liu Y, Yang Y (2020). The m6A reader YTHDF1 promotes ovarian cancer progression via augmenting EIF3C translation. Nucleic Acids Res.

[7] Wu Y, Wang Z, Han L, Guo Z, Yan B, Guo L (2022). PRMT5 regulates RNA m6A demethylation for doxorubicin sensitivity in breast cancer. Mol Ther.

[8] Zaccara S, Ries RJ, Jaffrey SR (2019). Reading, writing and erasing mRNA methylation. Nat Rev Mol Cell Biol.

[9] Huang H, Weng H, Chen J (2020). m(6)A Modification in Coding and Non-coding RNAs: Roles and Therapeutic Implications in Cancer. Cancer Cell.

[10] Guan Q, Lin H, Miao L, Guo H, Chen Y, Zhuo Z (2022). Functions, mechanisms, and therapeutic implications of METTL14 in human cancer. J Hematol Oncol.

[11] Liu L, Wang J, Sun G, Wu Q, Ma J, Zhang X (2019). m(6)A mRNA methylation regulates CTNNB1 to promote the proliferation of hepatoblastoma. Mol Cancer.

[12] Jiang X, Stockwell BR, Conrad M (2021). Ferroptosis: mechanisms, biology and role in disease. Nat Rev Mol Cell Biol.

[13] Lei G, Zhuang L, Gan B (2022). Targeting ferroptosis as a vulnerability in cancer. Nat Rev Cancer.

[14] Tang D, Chen X, Kang R, Kroemer G (2021). Ferroptosis: molecular mechanisms and health implications. Cell Res.

[15] Shi Z, Naowarojna N, Pan Z, Zou Y (2021). Multifaceted mechanisms mediating cystine starvation-induced ferroptosis. Nat Commun.

[16] Wu J, Minikes AM, Gao M, Bian H, Li Y, Stockwell BR (2019). Intercellular interaction dictates cancer cell ferroptosis via NF2-YAP signalling. Nature.

[17] Franklin JM, Wu Z, Guan KL (2023). Insights into recent findings and clinical application of YAP and TAZ in cancer. Nat Rev Cancer.

[18] Gao R, Kalathur RKR, Coto-Llerena M, Ercan C, Buechel D, Shuang S (2021). YAP/TAZ and ATF4 drive resistance to Sorafenib in hepatocellular carcinoma by preventing ferroptosis. EMBO Mol Med.

[19] Wang H, Lu J, Mandel JA, Zhang W, Schwalbe M, Gorka J (2021). Patient-Derived Mutant Forms of NFE2L2/NRF2 Drive Aggressive Murine Hepatoblastomas. Cell Mol Gastroenterol Hepatol.

[20] Tao J, Calvisi DF, Ranganathan S, Cigliano A, Zhou L, Singh S (2014). Activation of beta-catenin and Yap1 in human hepatoblastoma and induction of hepatocarcinogenesis in mice. Gastroenterology.

[21] Wang J, Zhu Q, Li R, Zhang J, Ye X, Li X (2022). YAP1 protects against septic liver injury via ferroptosis resistance. Cell Biosci.

[22] Chen Y, Zhu G, Liu Y, Wu Q, Zhang X, Bian Z (2019). O-GlcNAcylated c-Jun antagonizes ferroptosis via inhibiting GSH synthesis in liver cancer. Cell Signal.

[23] DeNicola GM, Chen PH, Mullarky E, Sudderth JA, Hu Z, Wu D (2015). NRF2 regulates serine biosynthesis in non-small cell lung cancer. Nat Genet.

[24] Du H, Zhao Y, He J, Zhang Y, Xi H, Liu M (2016). YTHDF2 destabilizes m(6)A-containing RNA through direct recruitment of the CCR4-NOT deadenylase complex. Nat Commun.

[25] Li H, Wolfe A, Septer S, Edwards G, Zhong X, Abdulkarim AB (2012). Deregulation of Hippo kinase signalling in human hepatic malignancies. Liver Int.

[26] Moroishi T, Hayashi T, Pan WW, Fujita Y, Holt MV, Qin J (2016). The Hippo Pathway Kinases LATS1/2 Suppress Cancer Immunity. Cell.

[27] Gan W, Dai X, Dai X, Xie J, Yin S, Zhu J (2020). LATS suppresses mTORC1 activity to directly coordinate Hippo and mTORC1 pathways in growth control. Nat Cell Biol.

[28] Yang J, Lee Y, Hwang CS (2023). The ubiquitin-proteasome system links NADPH metabolism to ferroptosis. Trends Cell Biol.

[29] Yang WH, Lin CC, Wu J, Chao PY, Chen K, Chen PH (2021). The Hippo Pathway Effector YAP Promotes Ferroptosis via the E3 Ligase SKP2. Mol Cancer Res.

[30] Bayir H, Dixon SJ, Tyurina YY, Kellum JA, Kagan VE (2023). Ferroptotic mechanisms and therapeutic targeting of iron metabolism and lipid peroxidation in the kidney. Nat Rev Nephrol.

[31] Marchesani F, Zangelmi E, Murtas G, Costanzi E, Ullah R, Peracchi A (2023). L-serine biosynthesis in the human central nervous system: Structure and function of phosphoserine aminotransferase. Protein Sci.

[32] Wang J, Zeng L, Wu N, Liang Y, Jin J, Fan M (2023). Inhibition of phosphoglycerate dehydrogenase induces ferroptosis and overcomes enzalutamide resistance in castration-resistant prostate cancer cells. Drug Resist Updat.

[33] Choi BH, Rawat V, Hogstrom J, Burns PA, Conger KO, Ozgurses ME (2022). Lineage-specific silencing of PSAT1 induces serine auxotrophy and sensitivity to dietary serine starvation in luminal breast tumors. Cell Rep.

[34] Wang H, Cui L, Li D, Fan M, Liu Z, Liu C (2020). Overexpression of PSAT1 regulated by G9A sustains cell proliferation in colorectal cancer. Signal Transduct Target Ther.

[35] Montrose DC, Saha S, Foronda M, McNally EM, Chen J, Zhou XK (2021). Exogenous and Endogenous Sources of Serine Contribute to Colon Cancer Metabolism, Growth, and Resistance to 5-Fluorouracil. Cancer Res.

[36] Yang CS, Stampouloglou E, Kingston NM, Zhang L, Monti S, Varelas X (2018). Glutamine-utilizing transaminases are a metabolic vulnerability of TAZ/YAP-activated cancer cells. EMBO Rep.

[37] Gao S, Ge A, Xu S, You Z, Ning S, Zhao Y (2017). PSAT1 is regulated by ATF4 and enhances cell proliferation via the GSK3beta/beta-catenin/cyclin D1 signaling pathway in ER-negative breast cancer. J Exp Clin Cancer Res.

[38] He XY, Fan X, Qu L, Wang X, Jiang L, Sang LJ (2023). LncRNA modulates Hippo-YAP signaling to reprogram iron metabolism. Nat Commun.

[39] Dey A, Varelas X, Guan KL (2020). Targeting the Hippo pathway in cancer, fibrosis, wound healing and regenerative medicine. Nat Rev Drug Discov.

[40] Nicholson AL, Pasquinelli AE (2019). Tales of Detailed Poly(A) Tails. Trends Cell Biol.

