# Supplementary material for: N6-methyladenosine modification of LATS2 promotes hepatoblastoma progression by inhibiting ferroptosis through the YAP1/ATF4/PSAT1 axis
Source: Int J Biol Sci. 2024 Aug 1;20(11):4146–61. doi: 10.7150/ijbs.92413 (PMC11379071; doi:10.7150/ijbs.92413)
Supplement: Supplementary file 1 — Supplementary figures and table. [file ijbsv20p4146s1.pdf]

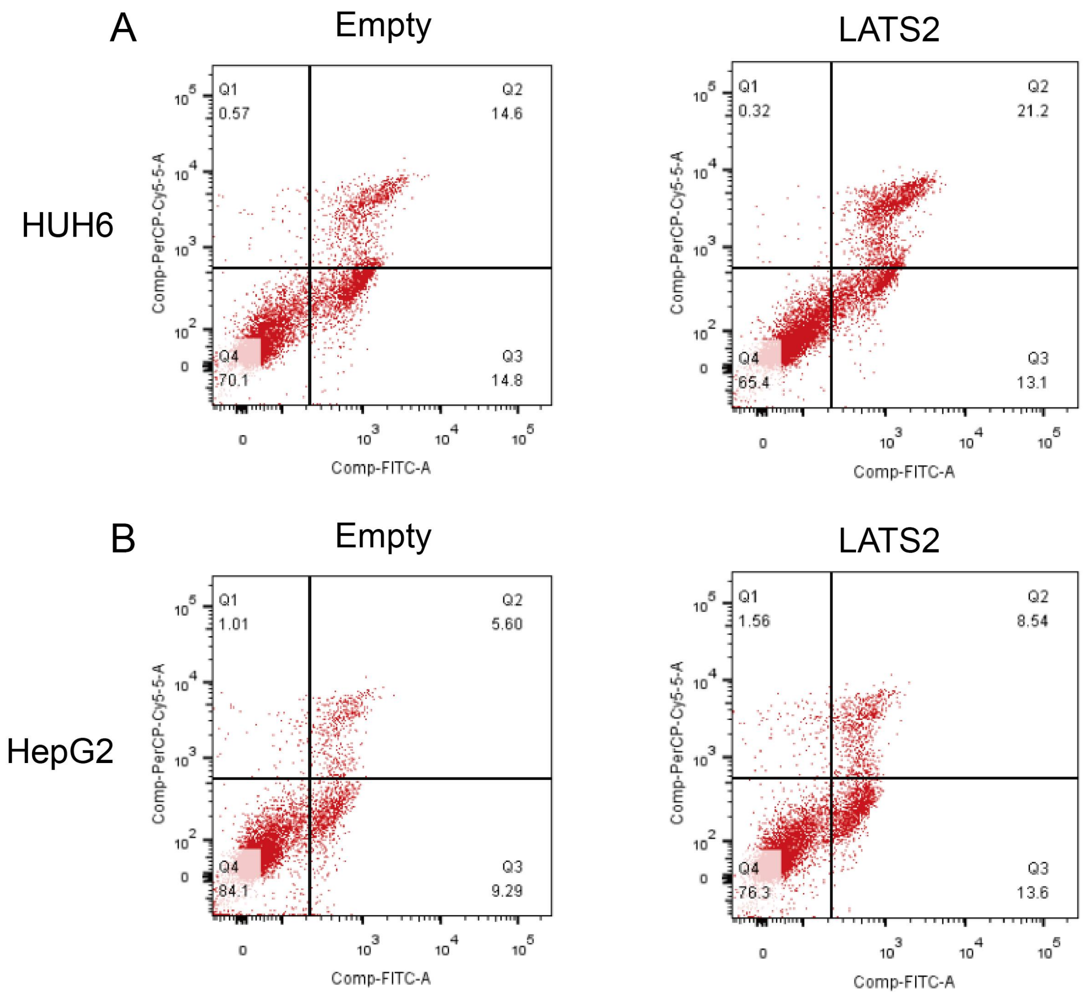

Supplementary Figure 1: LATS2 overexpression has negative influence on HB cell proliferation.

(A and B) Flow cytometry was used to evaluate the extent of apoptosis induced by LATS2 overexpression in HUH6 (A) and HepG2 (B) cells. All quantitative data are shown as the mean ± SD from three independent experiments.

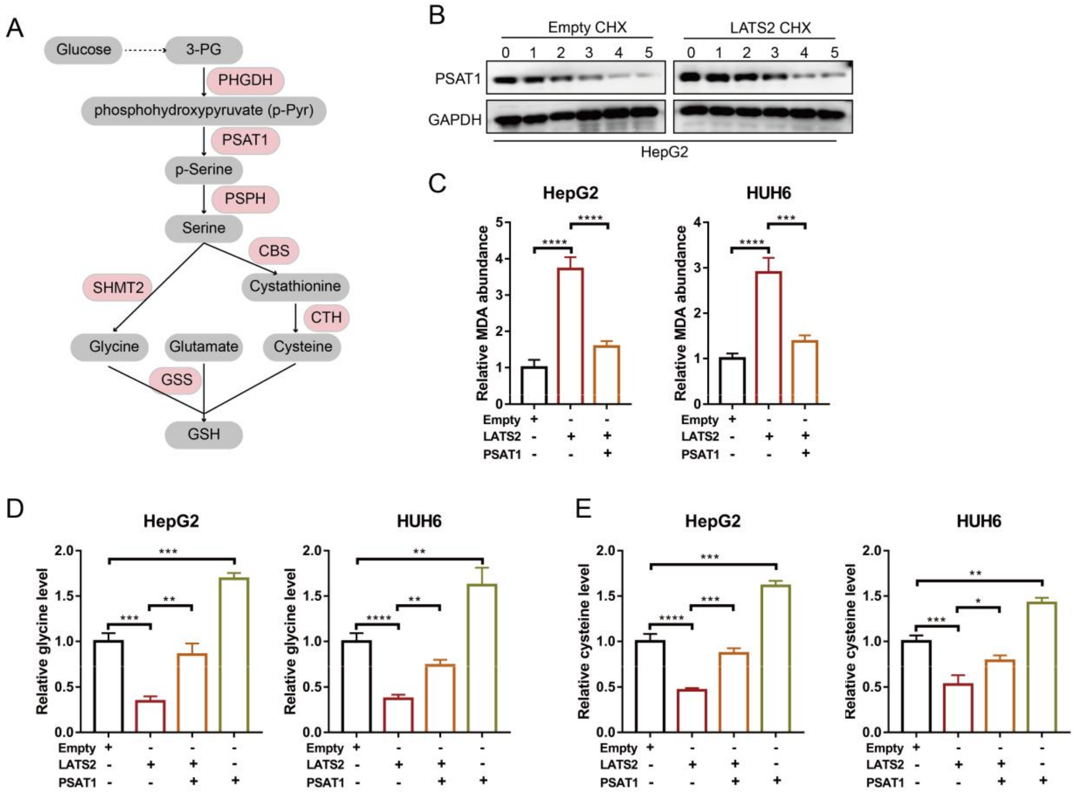

Supplementary Figure 2: LATS2-induced ferroptotic events are mediated by PSAT1. (A) Mechanistic diagram of the serine synthesis pathway. (B) Cycloheximide (CHX) chase experiments showing the half-life of PSAT1 after LATS2 overexpression in HepG2 cells at the indicated times after the addition of CHX (at a final concentration of 0.1 mg/ml). (C) MDA production was measured in HepG2 and HUH6 cells with simultaneous LATS2 overexpression and PSAT1 overexpression. (D and E) Relative levels of glycine (D) and cysteine (E) were measured in HepG2 and HUH6 cells under the indicated conditions. All quantitative data are shown as the mean ± SD from three independent experiments. *p <0.05, **p <0.01, ***p <0.001, ****p <0.0001

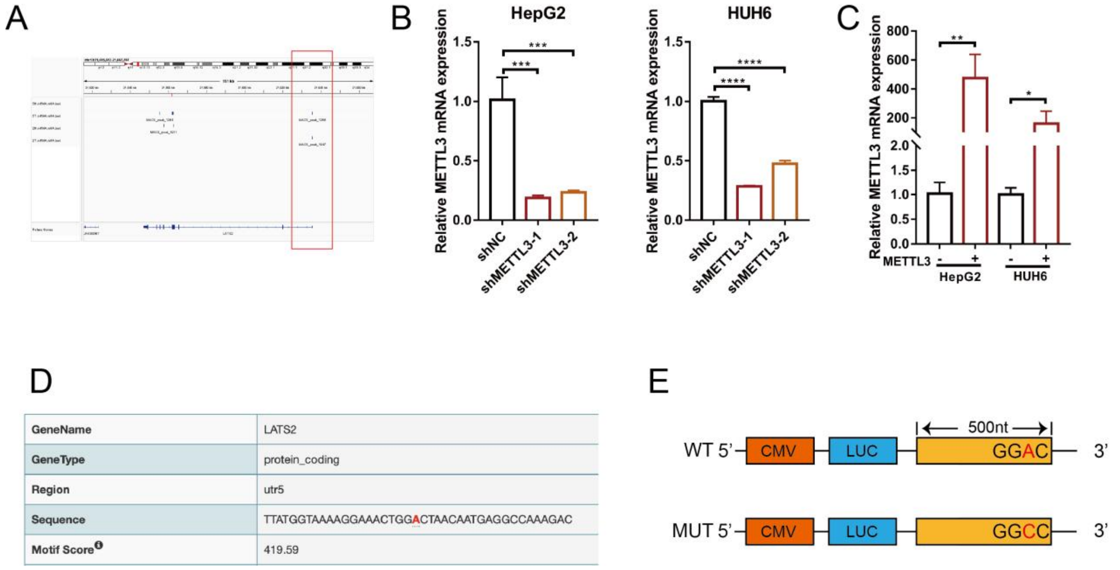

Supplementary Figure 3: LATS2 mRNA is modified by m6A methylation.

(A) The m6A peaks within LATS2 mRNA revealed by MeRIP-seq. (B and C) The mRNA level of METTL3 was verified by qRT–PCR after METTL3 knockdown (B) or overexpression (C). (D) The putative m6A site of LATS2 was predicted based on RMBase v2.0. (E) Schematic representation of the pmir-GlO luciferase reporters containing WT and Mut (GGAC to GGCC) LATS2 mRNA 5′ UTRs. All quantitative data are shown as the mean ± SD from three independent experiments. *p <0.05, **p <0.01, ***p <0.001, ****p <0.0001

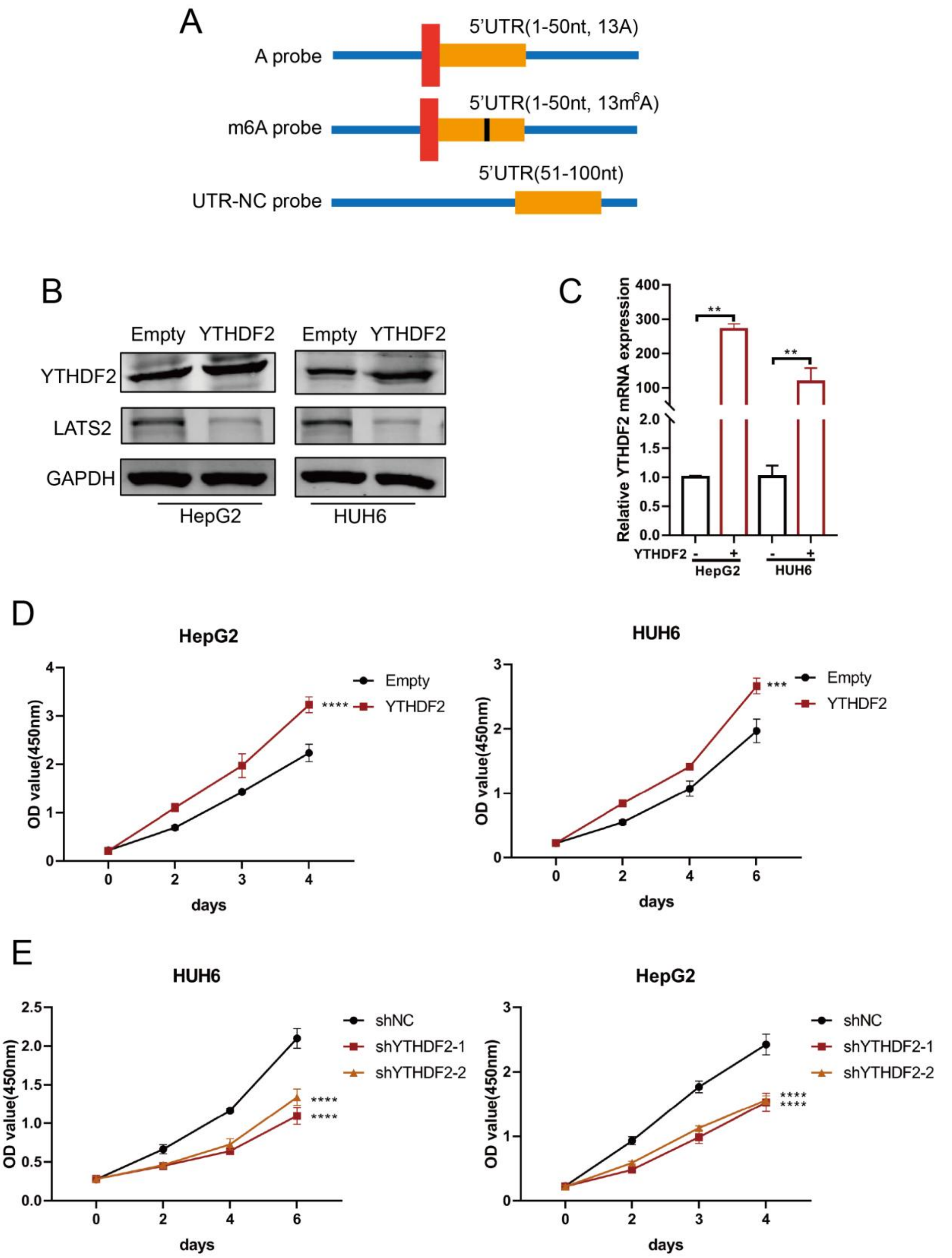

Supplementary Figure 4: YTHDF2 recognizes the m6A modification of LATS2 mRNA and facilitates the proliferation of HB cells.

(A) Schematic representation of the LATS2 probes with methylated or unmethylated adenosine used for screening putative m6A readers. (B and C) The YTHDF2 overexpression efficiency in HepG2 and HUH6 cells was verified by WB (B) and qRT–PCR (C). (D and E) CCK-8 assays were conducted to evaluate the role of YTHDF2 in the proliferation of HepG2 and HUH6 cells in which YTHDF2 is overexpressed (D) or knocked down (E). All quantitative data are shown as the mean ± SD from three independent experiments. n.s., no significant difference, *p <0.05, **p <0.01, ***p <0.001,

****p <0.0001

Supplementary Table 1: Bioinformatics analysis using the ChIP-seq dataset GSE99315 to confirm multiple potential YAP1 binding sites in the PSAT1 promoter region
PSAT1, NM_058179-promoter
ATGTGCAGTGGCCGCGTGCACCTGTGCCACAAATGGACAGGTGAACTCAATTTGCCA
CAGTTCCCACTACTTGTTTCATATCCCTGAGTGGCCCCTGGAGGCATTTGAGTTTGTGG
CTCTGGTTTAAGGTGTTTGTGGGCACAGTGTGAGGAAGAAACATGGAAAAGACAGATT
TTCTCTAGACTGAAAAGGAGATTGCCCAGGGGCGGGAGGAAGACAAACAGAGGTCAG
TGGGTCCTGAGGCTGACTGTATGTGTGACTTGTGTCCCTGAAATACCATCTTGGAAACT
GCAGGACCCCCGGGAGGAATGGCTGCAGGGGATGTCTTAGCAGATGAGACAATAGC
CACCGCCACCCCACCCCAAATTCCTGTGCCCCTAGTGGGATACAGAAGTAGTAGGTT
GCTCATCAACCCAAGCAGCCACATCAGCTTGGGCAGTGGAAACAACTCAGCCATATCT
TTTGGGAACAGAGGACCAAATGGATGTGCTGTCCCTTCTCCCAACCCACTACATGGGA
CTGTGTATAGCCCTGGTGTTAGGAACTAACTCCAGGAAGGATGAAGGCTGACTCCCTT
AGTCTCCAGTAGATAAGCTGCTAGGGGCAGCTACTAATATATAATAACTGAGTTATTTA
CGTAAAATAATGGATATGTGATGCTTCTTGCATGCCTGAGTTTCTGGGCTGAGATTTATT
CTGTCTTGAATGTCGCGGTTTTCTTAATGAAGTTGCTGAGGAACGCAGGGGCCTTGTT
CATTTTGCCTTTTTCTGGAAACTTTGTCCTCCAATTCCCAGATCCAGAGCAGTGCCTCT
GCTCTCAGCCCCATACCGCCCACATTCTCATTAGGAAAAGCAAACAAAAGCCAAAGTC
CTGTCCAGTTACAAGCCTTCTCTAAACGGGGCAGTTGGACTGTATATATTCCTGGCGC
ATCAATTTTACTCAGACAGGGAAAATATTTTTACATTAAAAGAAACTAGGTTAAATTATGG
TAGAGAATGCAAAATTCACAGTTTAAAAATGATGAAATTCCAGACTTCAAAGGAACTTCT
TTCTTGCAGGGTAGGGGGAGGGTATTCTGTTTCAGAACCCCATGCGGGTCTCCACTG
GAGTTCTTTTGAGAAGGACACTTCTGTGGAAAAGTTTGAGCAGCTCTGGCCCTGCGCC
TGGCCTGGCTAGGGGCCACCTTCTTCTGGTTTGGGCTGCAGCGCCCTATGGGTATCG
CGTTCTTGTTAATATCTCCTCACGTTTCTAAACTCACAGCTTGTCAGCGCGGGCGCAAC
CTGAGAGCTGTCGCAGGTTTCCAGCTCACTCGTTTCCCAAAGGAGGAAATGGAGAATC
AGCGACTTTAAAGGACTTGCCTGGCGGGCATCCACGCTTCCCAGGCTATCGCTCCTC
CCTGCGTCCTTGGCCACCTCCGTTCTTTAATCCTGCAGGAACTCAGGACCCACGTGCA
AATACAAAGAACCGTATCCACCCACCCCCGGCCCTTCTTCATCCTGCGCTTCCAACCT
TGGGGGGGTCCTCTCTCTCCACTGGGGTATCTGACGCCCGTGGGGGGTGCTTAGAAA
AGTTTGGTAGATTAGTGACTGGTGGTTCTGGAACAAATACATCACAGCCCAAACTGGG
GGCTGGTGGTGGAGAGGTGGGTGGATGGGGGCTACAAATCTGCTCGGCAACTGCCC
TTTCAGCCAAGAGAGAGGAGCTGAGGTCCTCTGGGGTTGGAGGACTGGAACCGGCCA
GATTGCGGGCTCAAGGGGCGAAGGCAGGTTGGCAGGGGCAGCCTCTTCCCGCCGCC
CACAATCCTCGGGCGGGCGCGCAGCCGAGCCGGCTCGGCTGGCTGGCGCAATCTC
GCGCGCTCCTTGCATTGATCAAAAATGGGGGTTGAAACAGTAAACGCGAGGAGGAGC
AACTGCTTCGACTCGGCTCAGAAGCGCGACCAATGGGGATGTGAGCTCCTTCGCGCG
AACCAATTAGCGCAGGGCCTGCGACAGCACGGGCCAATGGGGCGCCGACTCGGCGC
AGGAACAAGGCGGGGGTTCGGGGCCGGCTGCAGACTCTCACCGCAGCGGCCAGGA
ACGCCAGCCGTTCACGCGTTCGGTCCTCCTTGGCTGACTCACCGCCCTGGCCGCCG
CACC

Matrix ID	Name	Score	Relative score	Sequence ID	Start	End	Strand	Predicted sequence
MA0415.1	MA0415.1.YAP1	13.159885	0.8719493837922803		628	647	+	AGTTATTTACGTAAAATAAT
MA0415.1	MA0415.1.YAP1	10.151334	0.8292663518314142		628	647	-	ATTATTTTACGTAAATAACT
MA0415.1	MA0415.1.YAP1	4.3293214	0.7466680867430571		1557	1576	+	GGGTGCTTAGAAAAGTTTGG
MA0415.1	MA0415.1.YAP1	3.612979	0.7365051672577918		580	599	+	TCTCCAGTAGATAAGCTGCT
MA0415.1	MA0415.1.YAP1	2.6781514	0.7232425478246937		838	857	+	TTCTCATTAGGAAAAGCAAA

http://jaspar.binf.ku.dk/illustration;

The default Relative score is 0.8 (below 0.7, we consider no combination). Strand means that the positive and negative strands bound to DNA, both positive and negative strands are valid combinations. The larger the score, the higher the likelihood of combination, and greater than 5 points is considered a better combination.
